# Pelvic Organ Prolapse Repair with and without Concomitant Burch Colposuspension in Incontinent Women: A Randomised Controlled Trial with at Least 5-Year Followup

**DOI:** 10.1155/2012/967923

**Published:** 2011-10-19

**Authors:** Elisabetta Costantini, Massimo Lazzeri, Vittorio Bini, Michele Del Zingaro, Emanuela Frumenzio, Massimo Porena

**Affiliations:** ^1^Section of Urology and Andrology, Department of Medical-Surgical Specialties and Public Health, University of Perugia, 06100 Perugia, Italy; ^2^Department of Internal Medicine, University of Perugia, 06100 Perugia, Italy

## Abstract

The aim of this study was to reevaluate and update the followup of a previously published randomized controlled trial (RCT) on the impact of Burch Colposuspension (BC), as an anti-incontinence procedure, in patients with UI and POP, who underwent POP repair. Forty-seven women were randomly assigned to abdominal POP repair and concomitant BC (24 patients; group A) or POP repair alone without any anti-incontinence procedure (23 patients; group B). Median followup was 82 months (range 60–107); from over 47 patients, 30 reached 6-year followup. Two patients were lost at followup. In group A, 2 patients showed a stage I rectocele. In group B, 2 patients had a stage I rectocele and 1 a stage II rectocele. In group A, 13/23 (56.5%) were still incontinent after surgery compared with 9/22 patients (40.9%) in group B (*P* = 0.298). No significant changes were observed between the first and the current followup. The update of long-term followup confirmed that BC did not improve outcome significantly in incontinent women when they undergo POP repair.

## 1. Introduction

The prevalence of POP and UI increases with aging, and their association is not negligible [1–3]. Urologists and gynecologists have long performed POP repair in concomitance with an anti-incontinence procedure in women with SUI [4]. For many years, the concomitant anti-incontinence gold standard procedure was the BC. Studies with short- and midterm followups doubted the benefit of concomitant BC and POP repair in women with *or without* UI [5–8].

Thus, rigorous scientific assessment of the long-term outcome of surgery for POP and UI is essential in order to get an idea of what we are achieving in this field of non-life-threatening disease. It can be obtained by extending followup. Long-term followup is the main factor affecting the validity of interstudy comparisons of the efficacy and safety of treatment for POP and UI repair.

In order to evaluate the long-term impact of BC as an anti-incontinence measure during abdominal POP repair in patients with concomitant UI, we updated the followup of a RCT, whose midterm outcome *(three-year F-U)* has already been published [8]. 

## 2. Material and Methods

### 2.1. Study Population

The study design, inclusion and exclusion criteria were described in detail in previous paper [8]. To sum up, from January 2002 to June 2006, 47 women suffering from POP and *Stress UI* were randomly assigned to abdominal POP repair and concomitant BC (24 patients; group A) or POP repair alone without any anti-incontinence procedure (23 patients; group B). 

All patients were assessed by means of history, clinical examination, UDI-6 and IIQ-7 questionnaires, bladder diary, urine culture, 1 h pad test, and pelvic ultrasound. Vaginal inspection was performed in the gynecological and standing positions, at rest and under maximum straining with a full bladder. *POP was evaluated according to POP-Q systems* for quantitative description of POP. Clinical neurological tests of the perineum and lower limbs were normal in all patients. Urinary symptoms were recorded according to ICS criteria [9] and split into *voiding* and *storage* symptoms. All patients underwent urodynamic assessment, complying with ICS standards, which consist of uroflowmetry, filling cystometry, urethral pressure profile, pressure-flow study, and the VLPP [10]. UI was classified clinically on the basis of the ICS definition and graded on the Ingelman Sundberg scale [11]. Before and after prolapse repositioning all patients underwent a stress test in the supine position at maximum physiological bladder capacity. Patients with a negative stress test (also called cough test) when prolapse is not corrected but with a positive stress test when the prolapse is corrected (masked incontinence) were considered positive.

A standard Burch procedure, *using non-reabsorbable suture,* was performed as originally described [12]. The abdominal sacropexy was performed as previously reported [13, 14]. After entering the peritoneal cavity, the anterior vaginal wall was dissected from the bladder as far the bladder neck to expose a vaginal wall area of at least 3–5 cm where the mesh was attached with four polyglycolic 0 sutures. The posterior vaginal wall was freed as far as the * levator ani* plane and the mesh were attached with four polyglycolic 0 sutures. The two polypropylene prostheses were tailored and after the sacral promontory surface was prepared were placed in the sacral periosteum, about 2 cm below the promontory by one or two non-reabsorbable 00 sutures, avoiding excessive traction. The peritoneum was closed over the meshes. When the uterus sparing surgery was performed, two proximal sutures were positioned on the anterior and posterior cervical areas. The two polypropylene prostheses were cut: one rectangular and one Y shaped; the right and the left edges of the anterior Y-shaped mesh were passed through the broad ligaments, at a nonvascular point about 1 cm from the external part of the isthmus. Sacropexy or the hysterosacropexy was the first surgical step and BC the second step.

### 2.2. Randomization Process

Patients were assigned to groups according to a randomized computer-generated block design provided by the Statistics Department of the University of Perugia. Participants were intentionally allocated in equal numbers to each intervention according to a randomization ratio 1 : 1. *Patients were masked to Burch allocation, and investigators who operated were not blinded to group assignment.*

### 2.3. Followup

Check-ups were scheduled at 3, 6, 9 months postoperatively and then annually. They included a detailed urogynaecological history, clinical examination, and stress test. Between September and December 2010 all patients' charts were reevaluated to update followups. 

The primary outcome measures were (1) changes in the continence status (including urgency and SUI) as indicated by bladder diary, number of daily pads, and stress test. Success was defined as a completely dry patient: no leakage reported in the bladder diary, no pad use, and a negative stress test; (2) anatomical outcome of prolapse repair. Objective success was defined as the cervix/vault remaining well supported >6 cm above the hymen plane and no vaginal prolapse greater than or equal to *stage* 2 at any vaginal site while the patient performed a Valsalva maneuver. The secondary endpoints were changes in subjective symptoms and QoL as measured by questionnaires (IIQ-6, UDI-7). A VAS score (0–10) was recorded to assess the postoperative satisfaction (0 corresponded to the lowest satisfaction and 10 to the highest satisfaction with surgery). Finally, all patients were asked: “Would you repeat the operation again?” Not-masked research staff people performed the followup evaluation. 

The study was registered at http://www.clinicaltrials.gov/ (Protocol ID: NCT00576004) [8]. 

 Preliminary power analyses indicated that the samples of 47 patients provided statistical power (1-*β*) of at least 80% at *α* = 0.05 for detecting 35–40% in differences of proportion of postoperative incontinence between the two groups, when the incidence of postoperative conditions in group B equaled 10–30%, respectively. The Mann-Whitney test and Wilcoxon test for paired data were used to compare ordinal and nonnormally distributed continuous variables (deviation from Gaussian distribution was checked by the Kolgomorov-Smirnov test). Categorical data was analyzed by the McNemar test, *X* ^2^ test, or Fisher's exact test. Significance was set at *P* < 0.05. Data were analyzed using PASW release 17.0.2, SPSS Inc., Chicago, USA, 2009. 

## 3. Results

The overall median followup was 82 (range 60–107) (group A: 82 (range 61–107) and group B: 80 (range 60–100)). 

The two groups (A-B) did not differ significantly in any demographic and clinical variable (Table 1). Urodynamic and perioperative complications were described in a previous paper [8]. Two patients were lost at followup: one in group A committed suicide; one in group B moved abroad and did not attend followups anymore. 

In group A 2 patients showed a stage I rectocele. In group B 2 patients had a stage I rectocele and 1 a stage II rectocele. Incontinence persisted in 13/23 patients (56.5%) in group A and in 9/22 (40.9%) in group B (*P* = 0.657). No significant changes in incontinence status, voiding and storage symptoms emerged since midterm outcomes [8].  Table 2 reported all data comparing medium and long-term followup. As regards changes of continence status we did not find a significant increase of incontinent patients at 5-year followup: 56.5% of patients in group A were incontinent (versus 54.2% at mid term followup) and 40.9% of group B (versus 39.1 at midterm followup). Four patients, with persistent SUI, underwent midurethral slings and were dry at the last followup (Table 2). 

All group A patients were successfully treated for voiding dysfunction except for one who developed a rectocele after 5 years and showed recurrence of voiding symptoms. One patient, who had been considered cured of storage symptoms [8], developed them again (Table 2). 

In group B no changes were recorded in voiding dysfunction since the previous followup. One patient, who had been considered cured of storage symptoms [8], developed them again (Table 2). One patient had developed *de novo* storage symptoms and one had become incontinent since the previous followup. The results of incontinence treatments are illustrated in Table 2. Two patients with persistent SUI underwent midurethral slings and were dry at the last followup. 

Table 3 summarized the followup for the other parameters. Anatomical results had not changed since the previous followup. Questionnaires and the VAS score at long-term followups showed excellent results. 

## 4. Discussion

To date no consensus has as yet been reached on whether or not an anti-incontinence procedure should be performed concomitantly with POP repair, independently of the presence or absence of urinary incontinence. 

As far as regarding patients with preoperative SUI, BC might change outcomes, when associated with an abdominal POP repair, as it will act upon the bladder and urethra to improve continence. In order to answer we randomized 47 women with POP and UI to abdominal POP repair with concomitant BC or POP repair alone [8]. At a midterm followup, BC did not provide any additional benefit in incontinent patients as 54.2% were still incontinent after POP with BC compared with 39.1%, who did not receive BC (*P* = 0.459) [8]. The present long-term followup confirms midterm outcome. At a 5-year followup, BC is associated with higher incontinence rate, as previously reported, and does not add any benefit to urinary function. Our findings seem to be in line with data by Cosson et al. who reported that only 34% of the patients with prolapse and preoperative SUI achieved complete correction of the urinary dysfunction with a BC procedure during sacrocolpopexy [15]. When the Burch is combined with sacrocolpopexy results do not appear as good as those of an isolated Burch procedure which shows a long-term cure rate of 69% after 10–12 years. Cosson et al. retain that failure might be due to excessive traction on the anterior mesh and suggest performing colposuspension before colpopexy [15]. It is our opinion that abdominal colpopexy could be more at risk for postoperative SUI because of the change in the vaginal axis. Even though we have been performing sacropexy for years without excessive traction on the vaginal walls, in some predisposed patients (i.e., those with occult intrinsic sphincter deficiency) the direction of traction alone might be enough to cause SUI. Applying an adjunctive factor, such as the BC, with an anterior traction on the bladder neck, may increase the risk of SUI. Other factors such as surgical damage to urethral sphincter innervation and to the periurethral vascular plexus might also come into play. Other criticism may be advanced about BC. BC lengthens the operating time and may be a source of complications [16]. 

Recently a mail survey on members of International Urogynaecological Association investigated the practice patterns in the management of UI and POP [17]. The survey showed that most IUGA members perform BC as well as other procedures (i.e., TVT) for surgical therapy of urinary incontinence with genital prolapse. Borstad et al. compared the result of TVT performed at the time of prolapse repair or 3 months later in women with POP and SUI, by a multicenter prospective randomized trial [18]. From over 181 women with POP and SUI, 87 were randomized to have a TVT at the time of prolapse repair (group I) and 94 women 3 months later (group II). All the women in group II were evaluated for SUI 3 months after the prolapse repair and 53 women with confirmed SUI had a TVT performed. They found that 95% and 89%, respectively, for group I and II, were cured, but only 27% were cured after prolapse surgery alone. Although the study was well conducted and the results valid the followup was short: 1 year. Fujihara et al. reported their experience in 643 patients who underwent a TVM [19]. The aim of the study was to establish whether concomitant SUI and cystocele repair or two-stage surgery was the best solution for patients with cystocele. They showed that 37.9% of patients who were continent before surgery developed *de novo* incontinence and 6.3% underwent TOT 3 months later. On the other hand TVM cured only 29.7% of patients who were incontinent preoperatively and 6% needed a TOT after 3 months. The authors concluded by recommending the 2-stage approach. Unfortunately the followup is very short, and no long-lasting conclusion may be drawn.

Few studies have addressed changes in storage and voiding bladder symptoms after abdominal CSP with or without BC at a long-term followup. In women without SUI Burgio and colleagues found that preoperative storage and voiding symptoms improved independently of BC although BC seemed to reduce urgency [20]. In our series we found a significant improvement of symptoms throughout groups after surgery after a mean followup of 3 years. As we extended our mean followup to a minimum of 6 years with a median of 80 months, we did not find any significant differences or changes in the lower urinary tract symptoms whether patients received BC or not. 

The present study contains inherent weaknesses. There is admittedly a significant risk of a type II error, which means saying there is “no difference” when a difference exists. The sample size might appear grossly inadequate for the hypothesis understudy and we are, in fact, aware it is sufficient only to detect large differences that would be biologically unexpected given the hypothesis understudy. Finally, although surgeons were not blinded, the staff that evaluated outcome did not know what the type of surgery the patient had undergone. This form of blinding may serve to minimize the risk of detection bias, which is known as observer, ascertainment, or assessment bias. Consequently, we realize that any conclusions might be questionable. However, even though acknowledging the study limitations might prejudice publication, we believe that our results at the end of such a long followup make a worthy contribution to an ongoing debate on what to do when treating patients with concomitant UI and POP.

## 5. Conclusion

When we extended the followup of our randomized controlled trial, results cast doubts on whether BC should be performed during POP repair in incontinent women. We found that in incontinent women BC did not add significant improvement. Further studies remain mandatory in order to confirm our long-term results and to investigate alternative anti-incontinence procedures.

## Figures and Tables

**Table 1:** Demographic and clinical variables of groups A and B. Patient data.

Colposuspension	Yes (24 patients)	No (23 patients)	*P*
Age (years; mean/range)	60.0 ± 10.6 (35.1–79.0)	62.6 ± 12.8 (26.9–76.4)	NS
Menopause (*n*/%)	18 (75.0)	18(78.3)	NS
Previous urogynecological surgery	5 (20.8)	9 (39.1)	NS
Hysterectomy	5 (20.8)	8 (34.8)	NS
Prolapse repair	4 (16.7)	5 (21.7)	NS
SUI surgery	0 (0.0)	2 (8.7)	NS
BMI (kg/m^2^, median/range)	25.6 (20.8–35.2)	27.0 (16.0–31.9)	NS
Parity (median/range)	2 (0–3)	2 (1–3)	NS
ureterocele stage I	1 (4.2)	4 (17.4)	NS
ureterocele stage II	6 (25.0)	8 (34.8)	
ureterocele stage III	17 (70.8)	11 (47.8)	
Heart diseases (*n*/%)	2 (8.3)	1 (4.3)	NS
Hypertension (*n*/%)	6 (25.0)	8 (34.8)	NS
Hypercholesterolemy (*n*/%)	4 (16.7)	6 (26.1)	NS
Lower limb varices (*n*/%)	6 (25.0)	3 (13.0)	NS
COPD (*n*/%)	1 (4.2)	2 (8.7)	NS
Auto-immune disease (*n*/%)	2 (8.3)	3 (13.0)	NS
Anxiety (*n*/%)	5 (20.8)	6 (26.1)	NS
Diabetes mellitus type 2 (*n*/%)	2 (8.3)	3 (13.0)	NS
Recurrent urinary tract infections	2 (8.3)	3 (13.0)	NS
Hypothyroidism (*n*/%)	1 (4.2)	2 (8.7)	NS
Hydronephrosis (*n*/%)	1 (4.2)	0 (0.0)	NS

**Table 2:** Incontinence, voiding, and storage symptoms: long-term followup in groups A and B. Mid- and long-term outcomes.

	Group A				Group B			
	Previous followup: 50 months	Extended followup: 69 months	Treatment	Results	Previous followup: 46 months	Extended followup: 63 months	Treatment	Results
Incontinent patients	13/24	13/23			9/23	9/22		
	8 SUI	7 SUI			9 SUI	6 SUI		
	4 MUI	4 MUI				3 MUI		
	1 UUI	2 UUI						

Grade 1 UI	**6**	**6**	2 FKT (PFME)	Satisfied	**6**	**4**	3 no treatment	Satisfied
			4 no treatment	Satisfied with 1/pad die			1 antimuscarinic agents	Satisfied

Grade 2/3 UI	**7**	**7**	4 MUS	Dry	**3**	**5**	2 MUS	Dry
			1 refused surgery	Not satisfied			3 refuse surgery	Not satisfied
			2 antimuscarinic agents	Improved				

Voiding symptoms	Cured 17/17	Cured 16/17	No treatment		Cured 19/21	Cured 18/20	No treatment
					Improved 2	Improved 2	

	Cured 12/16	Cured 10/15			Cured 15/17	Cured 14/16		
Storage symptoms	Persistent 4	Persistent 5	2 antimuscarinic agents	Improved	Persistent 2	Persistent 3	2 antimuscarinic agents	Improved
	*De novo* 2	*De novo* 2			*De novo* 1	*De novo* 2		

**Table 3:** Secondary outcome end-points in patients of group A and B. Outcomes after mid- and long-term followups.

	Yes colposuspension (group A)			No colposuspension (group B)			Pre	Post
	Before (24)	*P*	After (23)	Before (23)	*P*	After (22)	A versus B group	A versus B group
Anatomical Results	24	<0.001	2	23	<0.001	3		
			2 stage I rectoceles			2 stage I rectoceles	—	NS
						1 Stage II rectocele		
No sexual intercourse	5	NS	7	10	NS	9	NS	NS
Disturbances during sexual intercourse	10	0.023	3	8	NS	4	NS	NS
No disturbances during sexual intercourse	9	NS	13	5	NS	9	NS	NS
Constipation	11	0.046	3 persistent	9	NS	2 persistent	NS	NS
			1 *ex novo *			1 *ex novo *		
IIQ7 (median/range)	16 (3–35)	<0.001	1 (0–11)	18 (1–45)	<0.001	2 (0–17)	NS	NS
UDI6 (median/range)	16 (6–45)	<0.001	3 (0–10)	16 (0–43)	<0.001	2.5 (0–14)	NS	NS
VAS (median/range)			8 (4–10)			8.5 (5–10)		NS
Patients would not repeat surgery			3			3		NS
Re-interventions for UI			4(MUS)			2 (MUS)		NS

VAS: Visual Analogue Scale.

MUS: Mediourethral sling.

## Abbreviation

BC: Burch colposuspension
B&W: Baden & Walker
CSP: Colposacropexy
ICS: International Consultation on Incontinence
IIQ: Incontinence impact on quality of life
PASW: Predictive Analytic Software
POP: Pelvic organ prolapse
POP-Q: Pelvic organ prolapse quantification
QoL: Quality of life
SUI: Stress urinary incontinence
TVM: Tension-free vaginal mesh procedure
TVT: Tension-free vaginal tape
UDI: Urogenital distress inventory
UI: Urinary incontinence
VAS: Visual analogue scale
VLPP: Valsalva leak point pressure.
